# Contrasting Becker and Duchenne muscular dystrophy serum biomarker candidates by using data independent acquisition LC-MS/MS

**DOI:** 10.1186/s13395-025-00385-3

**Published:** 2025-06-07

**Authors:** Camilla Johansson, Esther J. Schrama, David Kotol, Andreas Hober, Zaïda Koeks, Nienke M. van de Velde, Jan J. G. M. Verschuuren, Erik H. Niks, Fredrik Edfors, Pietro Spitali, Cristina Al-Khalili Szigyarto

**Affiliations:** 1 Department of Protein Science, School of Chemistry, Biology and Health, KTH Royal Institute of Technology, Stockholm, Sweden; 2 Department of Neurology, Leiden University Medical Center, Leiden, The Netherlands; 3 Science for Life Laboratory, School of Chemistry, Biology and Health, KTH Royal Institute of Technology, Stockholm, Sweden; 4 Department of Human Genetics, Leiden University Medical Center, Leiden, The Netherlands

**Keywords:** Becker muscular dystrophy, Disease progression biomarkers, DIA, SRM, Duchenne muscular dystrophy, Proteomics

## Abstract

**Background:**

Becker muscular dystrophy (BMD) is a rare and heterogeneous form of dystrophinopathy caused by expression of altered dystrophin proteins, as a consequence of in-frame genetic mutations. The majority of the BMD biomarker studies employ targeted approaches and focus on translating findings from Duchenne Muscular Dystrophy (DMD), a more severe disease form with clinical similarities but caused by out-of-frame mutations in the dystrophin gene. Importantly, DMD therapies assume that disease progression can be slowed by promoting the expression of truncated dystrophin comparable to what occurs in BMD patients. In this study, we explore similarities and differences in protein trajectories over time between BMD and DMD serum, and explore proteins related to motor function performance.

**Methods:**

Serum samples collected from 34 BMD patients, in a prospective longitudinal 3-year study, and 19 DMD patients, were analyzed by using Data Independent Acquisition Tandem Mass Spectrometry (DIA-MS). Subsequent normalization, linear mixed effects model was employed to identify proteins associated with physical tests and dystrophin expression in skeletal muscle. Analysis was also performed to explore the discrepancy between DMD and BMD biomarker abundance trajectories over time.

**Results:**

Linear mixed effects models identified 20 proteins with altered longitudinal signatures between DMD and BMD, including creatine kinase M-type (CKM) pyruvate kinase (PKM), fibrinogen gamma chain (FGG), lactate dehydrogenase B (LDHB) and alpha-2-macroglobulin (A2M). Furthermore, several proteins related to innate immune response were associated with motor function in BMD patients. In particular, A2M displayed an altered time-dependent decline in relation to dystrophin expression in the tibialis anterior muscle.

**Conclusions:**

Our study revealed differences in the serum proteome between BMD and DMD, which comprises proteins involved in the immune response, extracellular matrix organization and hemostasis but not muscle leakage proteins significantly associated with disease progression in DMD. If further evaluated and validated, these biomarker candidates may offer means to monitor disease progression in BMD patients. A2M is of particular interest due to its association with dystrophin expression in BMD muscle and higher abundance in DMD patients in comparison to BMD. If validated, A2M could be used as a pharmacodynamic biomarker in therapeutic clinical trials aiming to restore dystrophin expression.

**Supplementary Information:**

The online version contains supplementary material available at 10.1186/s13395-025-00385-3.

## Introduction

While biomarker research for Duchenne muscular dystrophy (DMD) [1–8] has increased over the past decades, less attention has been given to the milder form of dystrophinopathy, Becker muscular dystrophy (BMD) [1, 7]. Several of the biomarker studies have aimed at translating findings from DMD to BMD, assuming that these diseases share not only genetic features being caused by pathogenic mutations within the *DMD* gene, but also common clinical features. While the diagnosis of BMD is well established, biomarkers for monitoring disease progression and response to treatment are needed.

DMD and BMD, belong to the dystrophinopathy spectrum as they share the same pathogenic gene and characteristics such as progressive muscle degeneration and dilated cardiomyopathy. Disease symptoms appear during childhood in both diseases, but in BMD patients manifest for the first time also later in life during adulthood. The global prevalence of BMD is estimated to be 1.6 per 100,000 people, whereas that of DMD is 4.8 [9]. While DMD patients lose ambulation around their early teens [10], the BMD phenotype varies considerably between a slightly milder progression than DMD to a nearly asymptomatic state late in life [11]. Both DMD and BMD are caused by pathogenic mutations resulting in progressive muscle wasting, and cardiac and respiratory complications. The protein product of the *DMD* gene, dystrophin, is entirely absent or expressed at less than 3–5% of healthy expression in DMD patients [12, 13], most often due to out-of-frame variants in the gene. BMD patients often display in-frame variants and an increased but variable expression of partly functional dystrophin proteins in tissue (ranging from 5 to 100% of healthy expression) [14]. This variation in expression efficiency, along with the length and composition of dystrophin products are factors believed to contribute to the broad heterogeneity of disease severity in BMD. However, a considerable part of the variation in phenotype remains unexplained [11].

Furthermore, several DMD therapies currently in development are aimed at inducing a BMD-like phenotype. Exon-skipping and gene-therapy development for DMD patients assume that by increasing expression of partial dystrophin in tissue, DMD patients will experience a milder, more BMD-like phenotype with increased mobility and prolonged life expectancy. Currently, many dystrophin-restoring therapies that have gained accelerated approval by the FDA rely on dystrophin expression measurements [13]. Dystrophin expression is measured in muscle biopsies, which are not representative of the entire muscle. Due to their invasive nature, biopsies can only be collected in limited numbers and repetitions [15], enabling assessment of dystrophin expression in only a small area of a specific muscle [12]. Therefore, attention has been given to developing pharmacodynamic or disease progression monitoring blood biomarkers [3, 5, 16]. Monitoring biomarker candidates for DMD tend to have a longitudinal trajectory associated with patient age [1, 2, 6, 8] and it is assumed that these trajectories will change toward healthy trajectories upon increased partial dystrophin re-expression and reduced pathology. Additionally, the use of biomarkers as a surrogate endpoint in therapeutic trials in BMD would be a welcoming alternative for the currently used functional tests, which have a relatively low sensitivity to changes in disease progression [17, 18]. However, our understanding of longitudinal protein profiles in BMD patients is limited and few [19], if any, longitudinal BMD proteomic studies have been published to date.

In this study, we sought to explore the longitudinal proteomic similarities and differences between DMD and BMD patients and to identify biomarker candidates related to changes in motor function and dystrophin expression in BMD patients. Longitudinal serum samples from 34 BMD patients and 19 DMD patients were analyzed using liquid-chromatography mass spectrometry (LC-MS/MS) in data independent acquisition (DIA) mode. The study identified one protein that correlates with age in BMD patients as well as twenty proteins with either a faster or slower change with age in DMD compared to BMD patients.

## Methods

### Sample collection

BMD patients were recruited via the Dutch Dystrophinopathy Database for a 3-year prospective natural history study conducted at the LUMC between 2014 and 2019 [19]. The inclusion criteria were: male gender, age ≥ 18 years, and an in-frame variant in the *DMD-*gene or another variant in the *DMD*-gene with a mild phenotype (first symptoms at age ≥ 5 years). The participants were invited for 4 annual study visits. At each study visit, serum samples were collected and motor function performance was assessed through the North Star Ambulatory Assessment (NSAA), 10-meter walk/run test velocity (10MWRTv), rise from floor velocity (RFFv), and four stair climbing and descending velocity (4-SCv and 4-SDv respectively). The NSAA was also performed on non-ambulant participants to capture the full decline of the scale. These participants could obtain a maximum score of 6 points based on the items: stand tall (0–2 points), neck flexion (0–2 points) and sit from supine (0–2 points).

Serum samples were prepared as described by van de Velde et al. and stored at − 80° C prior to use [19]. Separate consent was obtained for a muscle biopsy from the right tibialis anterior (TA) in a subset of the participants. If the participant had also taken part in a natural history study for BMD conducted at the LUMC in 2011 (P10.133), samples obtained in that study were also included in the current analysis to extend the longitudinal observations in the BMD group previously [14]. Since clinical tests were not part of the 2011 study protocol, the clinical correlations in this paper are solely based on the 2014 study. Samples from DMD patients were obtained as part of the local biobank protocol P05.004. This protocol allowed the collection of serum samples as part of routine clinical care visits in all paediatric and adult patients with neuromuscular diseases and was approved by the LUMC ethical board in 2005.

### Serum sample preparation

A mixture of stable isotope labelled standards were prepared according to Table S1. Four microliters of standard mixture were dispensed into each well of a 96-well LoBind plate (0030129512, Eppendorf, Hamburg, Germany) and allowed to dry in a vacuum centrifuge according to a previously established protocol [20]. The dried standards were dissolved in 5 µl of denaturant buffer (9 M urea, 20 mM TCEP, and 1xPBS) and subsequently 5 µl of five times diluted serum was added to the standards. The samples were incubated at 37° C for one hour before 2-chloroacetamide was added to a final concentration of 75 mM. The samples were incubated for 30 min, shielded from light and subsequently diluted in 1xPBS to reach a final concentration of 0.5 M urea before the addition of 1.2 µg of trypsin (Sigma Aldrich, St Louis, MO, USA) per sample. The samples were incubated overnight (18 h) and the digestion was quenched by the addition of formic acid (FA) to a final concentration of 0.5%. The samples were desalted using StageTips, as described previously [21] and were resuspended in 0.1% FA prior to LC-MS/MS analysis.

### LC-MS/MS DIA

The DIA analysis was performed on an online system of Ultimate 3000 (Thermo Scientific) liquid chromatography connected to a Q Exactive HF (Thermo Scientific) mass spectrometer. Five micrograms of peptides were injected and washed for 1 min at 15 µl/min with Solvent A (3% ACN, 0.1% FA) on a trap column (PN: 160454, Thermo Scientific) and separated on a 15 cm analytical column (PN: ES806, Thermo Scientific) following a 50-min gradient of 1–32% Solvent B (95% ACN, 0.1% FA) at 3.6 µl/min. The washout was performed with 99% Solvent B for 30 s and two seesaw gradients of 1–99% Solvent B. Each DIA cycle comprised of one full MS scan at 60,000 resolution (AGC target 3e^6^, mass range 300–1,200 m/z and injection time 105 ms) followed by 30 DIA MS/MS scans with 10 m/z windows and 1 m/z margins ranging 350–1,000 m/z at 30,000 resolution (AGC target 1e6, NCE 26, injection time 55 ms). The acquired raw files were converted to mzML file format using ProteoWizard and the provided software tool MSConvert [22]. The resulting mzML files were searched in EncyclopeDIA [23] against a spectral library generated with Prosit [24]. The Prosit library was generated for sequences from 2064 proteins that were previously detected in blood plasma supplemented with 86 proteins previously proposed as potential DMD biomarkers. A whole human proteome (*Homo Sapiens* UniProt ID: #UP000005640, 20,371 entries, accessed 2021-08-11) was used as a background proteome in the library search.

### SRM-MS for panel of proteins

Approximately 10 µg of peptides were loaded onto an Ultimate 3000 LC-system (Thermo Fisher Scientific, Waltham, MA, USA) equipped with an Acclaim PepMap 100 trap column (PN 160454, particle size: 5 μm, pore size: 100 Å, 0.3 mm x 5 mm, Thermo Fisher Scientific) and a 15 cm EasySpray analytical column (PN ES802A rev.2, particle size: 2 μm, pore size: 100Å, 150 μm x 15 cm, Thermo Fisher Scientific). The LC-system was connected to a TSQ Altis (Thermo Fisher) mass spectrometer operating in a scheduled SRM mode with a cycle time of 1.0 s. The monitored transitions are specified in Supplementary file 1. The peptides were eluted from the LC-system using a linear gradient with a flow rate of 3 µl/min and a total method time of 35 min. The mobile phase consisted of solvent A (3% acetonitrile (ACN), 0.1% FA) and solvent B (95% CAN, 0.1% FA) and the gradient specifications are described in Table S2. All the raw files were analyzed in Skyline-daily (v. 19.1.1.309 (1dc16c97a)) [25].

### Data analysis

Proteins were inferred from the DIA data using EncyclopeDIA. Furthermore, proteins present in less than 95% of samples were removed (Fig. S1). Six normalization methods [26] were evaluated on their ability to reduce within- and between plate coefficients of variation, as calculated for nine replicates of a quality control sample per plate. The normalization methods tested were Total Ion Current (TIC), Median normalisation (Median), Probabilistic Quotient Normalisation (PQN), Median Absolute Deviation (MAD), quantile normalisation (Quantile) and bridge normalisation (BRDG). A combination of the two best performing normalisation methods were selected for all downstream analyses.

All statistical analysis was performed in R language version 4.1.0 [27]. Linear mixed effects models were performed using R package *lme4* [28]. All *P*-values were adjusted using the Benjamini-Hochberg method [29] to account for multiple hypothesis testing and displayed as false discovery rates (FDRs).

## Results

### Baseline characteristics

Thirty-six patients with BMD were included in the natural history study starting in 2014. Two participants were excluded one did not consent to serum sampling and one completed only a single visit for which serum sample was not available. The remaining 34 participants completed 123 study visits. Serum samples were missing or of insufficient quantity for analysis for 32 visits, resulting in 90 samples with a variable number of visits per participant: a single visit (4), two (6), three (23) or four (1) visits. Clinical data was missing on one occasion, resulting in 89 visits with both clinical data and an available serum sample that were included in the correlation with functional tests. For the longitudinal analysis, an additional sample from the 2011 study was available for 17 participants, taken 4.7 years (SD 0.9) prior to the first 2014 study visit, resulting in a total of 107 samples. Thirty-seven participants were ambulant at the first sample, one had lost ambulation between the visit for the 2011 study and the visit for the 2014 natural history study, and another three lost ambulation during the 2014 study.

Forty-eight samples of 19 DMD patients were collected. The number of samples per participant varied; one sample (5), two samples (5), three samples (6), four samples (1) or 5 samples (2). The mean follow-up in participants with multiple samples was 3.4 years (SD.2.1; range 1.0-6.9). Fifteen were non-ambulant at baseline. All four participants who were ambulant at baseline lost ambulation during follow-up. Eighteen participants were treated with an intermittent corticosteroid regime at the time of samples. One participant had been treated with corticosteroids in the past for short periods, but had not taken corticosteroids in the 6 months prior to the first sample. The baseline characteristics are shown in Table 1.

**Table 1:** Baseline characteristics

**DMD patients**	*N*	
Mean age (SD)	19	7.4 y (4.9)
Ambulant at baseline	4	21.1%
LoA during study	4	21.1%
Age at LoA (SD)	19	10.6y (2.1)
Genetic variants Deletion Duplication In-frame^†^ Out-of-frame Small variant	11 3 2 12 5	58.0% 15.8% 14.3% 85.7% 26.3%
**BMD patients**		
Mean age (SD) at first sample	34	39.9 y (12.1)
Ambulant at first sample	27	79.4%
Mean age (SD) at first functional assessment	34	42.3 y (12.4)
Ambulant at first functional assessment	26	76.5%
LoA during study	4	11.8%
Age at LoA (SD)	11	38.1 y (14.6)
Genetic variants Deletion Duplication In-frame Out-of-frame^††^ Small variant	26 5 29 2 3	76.5% 14.7% 93.5% 6.5% 8.8%
NSAA (SD)	33	16.7 (13.0)
10MWRTv (SD)	23	1.9 m/s (1.1)
4-SCv (SD)	20	1.5 steps/s (0.9)
4-SDv (SD)	20	1.7 steps/s (1.1)
RFFv (SD)	16	0.3 rises/s (0.1)
Dystrophin (SD)	13	43.3% (19.3)

Abbreviations: DMD = Duchenne Muscular Dystrophy; BMD = Becker Muscular Dystrophy; NSAA = North Star Ambulatory Assessment; 10MWRTv = 10 meter walk/run velocity; 4-SCv = 4-Stair Climb velocity; 4-SDv = 4-Stair Descend velocity; RFFv = Rise From Floor velocity. ^†^In-frame DMD variants were deletion of exons 3 to 18 and of exons 5 to 44. ^††^Both out-of-frame BMD variants were deletions of exons 3 to 7

### Study design and strategies for data preprocessing of DIA data

**Fig. 1:**
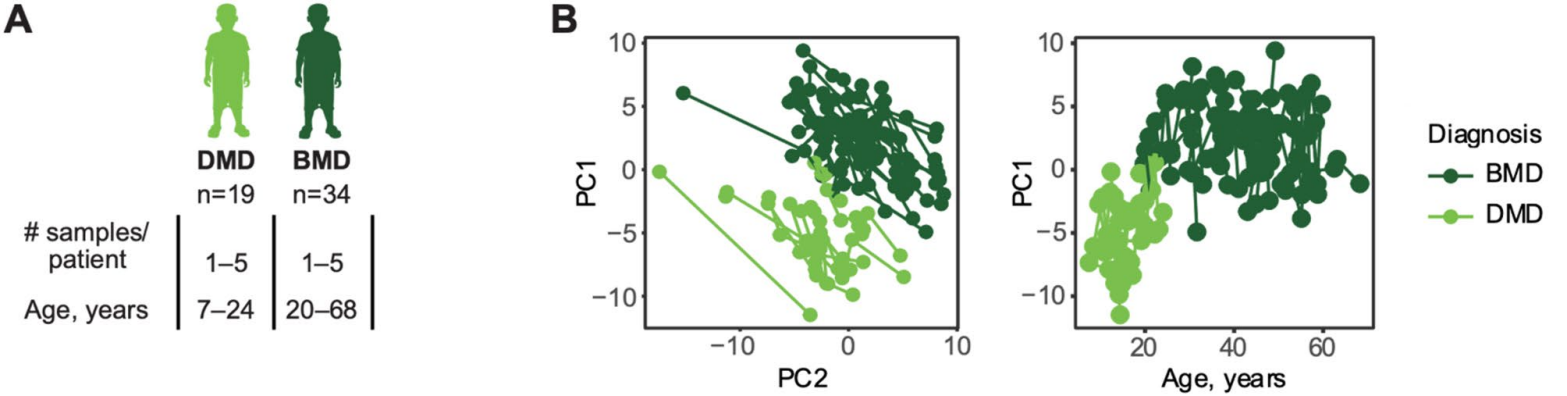
**A** Study design. **B** Principal component analysis on protein levels (left) separates DMD patients and BMD patients in first component. The first principal component (PC1) correlated with patient age (right). Each dot represents a sample and samples from the same patient are connected with a line. Light green indicates samples from DMD patients whereas dark green indicates samples from BMD patients

DIA mass spectrometry is a powerful tool for proteomic analysis, and its popularity has increased over the past years as more sophisticated algorithms have been developed to combat the challenges of peptide interference in compositional MS^2^ precursor spectra [30]. In this study, we used DIA MS to analyze 154 serum samples (Fig. 1A) with the aim of studying longitudinal differences between Becker muscular dystrophy (BMD) patients and Duchenne muscular dystrophy (DMD) patients and investigating potential biomarkers of dystrophin expression and functional outcome in BMD. A total of 250 unique proteins were identified from a Prosit spectral library of 2064 protein sequences.

To reduce the effects of technical variations, six normalization methods [26], Total Ion Current (TIC), Median normalisation (Median), Probabilistic Quotient Normalisation (PQN), Median Absolute Deviation (MAD), Quantille normalisation (Quantile) and Bridge normalisation (BRDG) were evaluated (Fig. S2). Probabilistic quotient normalization (PQN) [31] was found to best reduce within-plate and between-plate coefficient of variation (CV) (Fig. S2A-B), whereas principal component analysis (PCA) of quality control samples revealed that only total ion current (TIC) normalisation and within batch bridge normalisation (BRDG-WB) could reduce batch effects between sample replicates (Fig. S2C). PCA analysis of the whole cohort revealed that, while non-normalised data separated DMD and BMD patients in the third component, both median normalisation and PQN moved this separation to first or second component (Fig. S2D). PQN was originally developed for reducing variation from sample dilutions in NMR data [31] and has been used in both affinity proteomics and metabolomics for the same purpose [8, 26]. In metabolomics, PQN is preceded by the TIC normalisation [26]. Since our data contained proteins displaying extreme outlier behaviors (Fig. S3), likely due to keratin contamination, we reasoned that preceding PQN with a median or BRDG-WB normalisation would be more robust to protein outliers than the TIC normalisation. A combination of BRDG-WB and PQN was found to reduce both the batch effect and within plate variation and was therefore selected as the normalization method for all downstream analyses. As a quality control, the same investigation of normalisation methods was performed at peptide level, with comparable results (Fig. S4).

### Several proteins show different age trajectories between DMD and BMD

**Fig. 2:**
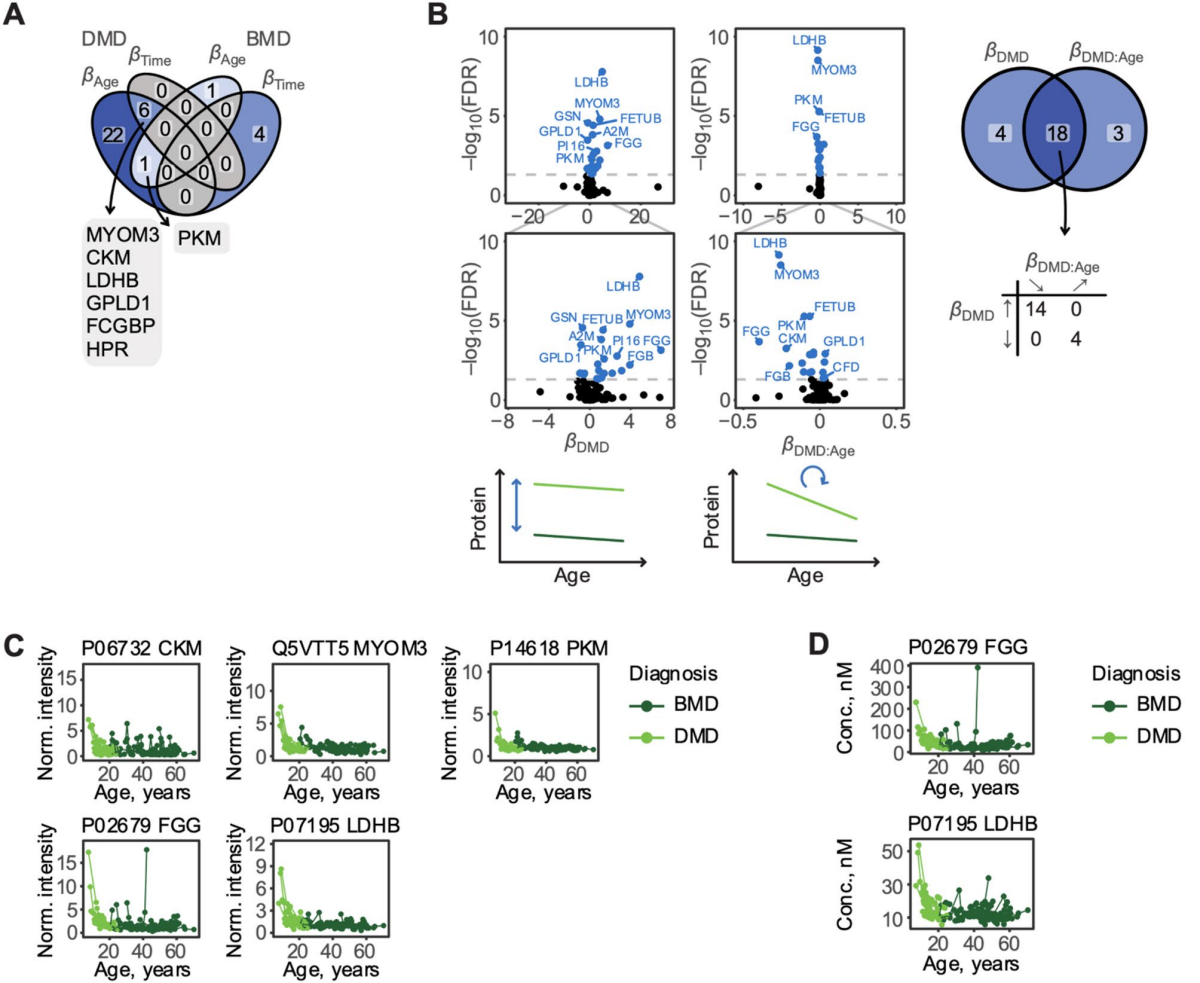
Proteins significantly correlated with age or time from first sample in DMD and BMD, separately, using linear mixed effects models with either age or time from first sample as fixed effect and patient as random effect. One protein (PKM) correlated with age in both DMD patients and BMD patients. **B** Volcano plots (left) for proteins that change in either intercept (*β*_DMD_) or trajectory slope (*β*_DMD: Age_) between DMD and BMD patients as assessed through a linear mixed effect model with age and diagnosis as fixed effects and patient as random effect. The Venn diagram (right) shows overlap between proteins that differ between DMD patients and BMD patients, with respect to both intercept and age trajectory. **C** Spaghetti plots for some proteins with changed age trajectory between DMD and BMD. Each dot represents a sample, and samples from the same patient are connected. **D** FGG and LDHB were absolute quantified and trajectories are confirmed using SRM-MS

Principal component analysis could almost completely separate DMD and BMD patients (Fig. 1B). Linear mixed effects models were used to assess whether differences between groups and age had a comparable role on the protein trajectories in DMD and BMD patients, i.e. detect changes in how fast a protein abundance in serum increased or declined with patient age. A total of 29 proteins were found to be correlated with age in DMD patients (Fig. 2A, Table S3) whereas two proteins correlated with age in BMD patients (Fig. 2A, Table S4), of which one protein, PKM, was associated with age in both DMD patients and BMD patients. While age is a good predictor of disease progression in DMD patients [8], it is less so for BMD, where progression is more heterogenous and less age dependent according to functional scales. Hence, we sought to investigate whether protein trajectories changed over time (up to seven years of follow-up) in DMD patients or BMD patients regardless of patient age and progression state at enrolment. Linear mixed effects models using time from the first collected sample as a fixed effect, showed that much fewer proteins changed over the course of the study compared to proteins that changed with patient age in DMD (Fig. 2C, Table S5). Four proteins changed over time in BMD (Table S6). These proteins showed significant associations only in BMD patients.

We then assessed whether the protein trajectories were comparable across DMD and BMD patients and identified a set of 21 proteins (Fig. 2B; Table 2) with altered age trajectory. We define an altered age trajectory as a significant change in the rate of decline or increase of protein abundance with age in DMD compared to BMD patients, *β*_DMD: Age_. A few proteins with altered age trajectories between DMD and BMD are shown in Fig. 2C. PKM, was associated with age in both DMD and BMD patients, but showed a steeper age trajectory in DMD patients. Using a previously developed and published absolute quantitative panel of heavy labelled protein epitope signature tags (PrESTs) [32], we confirmed the age trajectories of FGG and LDHB (Fig. 2D).

**Table 2:** Proteins with different age-trajectory between DMD patients and BMD patients. Significant FDR-values are marked with an * and in grey. The overlap between proteins that differ between DMD patients and BMD patients, with respect to both intercept and age trajectory, are listed in bold font

Uniprot ID	Protein name	Gene name	β _Intercept_	β _Age_	β _DMD_	β _Age: DMD_	FDR Intercept	FDR Age	FDR DMD	FDR Age: DMD
P07195	**Lactate dehydrogenase B**	**LDHB**	1.35	–0.008	4.88	–0.27	8.59E–04*	7.50E–01	1.65E–08*	7.24E–10*
Q5VTT5	**Myomesin 3**	**MYOM3**	2.06	–0.020	3.92	–0.26	7.54E–06*	3.03E–01	1.62E–05*	3.16E–09*
P14618	**Pyruvate kinase M1/2**	**PKM**	1.59	–0.013	1.40	–0.10	5.03E–10*	1.67E–01	2.55E–03*	5.32E–06*
Q9UGM5	**Fetuin B**	**FETUB**	1.18	–0.003	1.31	–0.07	4.09E–10*	7.50E–01	3.87E–05*	5.32E–06*
P02679	**Fibrinogen gamma chain**	**FGG**	2.30	–0.016	6.98	–0.40	1.30E–02*	7.70E–01	7.17E–04*	2.09E–04*
P06732	**Creatine kinase**,** M-type**	**CKM**	2.40	–0.028	3.12	–0.22	1.84E–05*	2.34E–01	1.41E–02*	5.56E–04*
P05019	Insulin like growth factor 1	IGF1	5.01	–0.070	–4.86	0.52	3.70E–03*	3.64E–01	2.98E–01	6.25E–04*
P01023	**Alpha-2-macroglobulin**	**A2M**	1.20	–0.004	1.12	–0.04	6.05E–11*	6.90E–01	1.53E–04*	9.77E–04*
P80108	**Glycosylphosphatidylinositol specific phospholipase D1**	**GPLD1**	0.99	0.002	–0.87	0.04	1.24E–11*	7.77E–01	3.32E–04*	1.21E–03*
P51884	**Lumican**	**LUM**	0.85	0.005	0.79	–0.04	2.34E–08*	4.76E–01	5.47E–03*	1.33E–03*
P11021	**Heat shock protein family A member 5**	**HSPA5**	1.29	–0.008	0.89	–0.05	1.54E–11*	2.98E–01	1.39E–02*	1.36E–03*
P22105	Tenascin XB	TNXB	1.63	–0.013	0.60	–0.07	2.61E–11*	1.67E–01	3.14E–01	1.36E–03*
P02743	**Amyloid P component**,** serum**	**APCS**	0.84	0.003	–0.52	0.03	4.63E–12*	5.43E–01	2.07E–02*	4.04E–03*
Q6UXB8	**Peptidase inhibitor 16**	**PI16**	1.45	–0.010	2.67	–0.12	6.69E–04*	6.70E–01	1.68E–03*	4.71E–03*
P02675	**Fibrinogen beta chain**	**FGB**	1.31	–0.004	3.93	–0.20	3.35E–02*	9.24E–01	6.11E–03*	6.87E–03*
P04278	**Sex hormone binding globulin**	**SHBG**	0.69	0.010	2.20	–0.11	1.15E–01	6.70E–01	2.04E–02*	1.69E–02*
P17066	**Heat shock protein family A member 6**	**HSPA6**	1.32	–0.008	0.90	–0.05	1.14E–09*	3.11E–01	4.53E–02*	1.69E–02*
P06396	**Gelsolin**	**GSN**	1.16	–0.002	–0.71	0.02	4.95E–21*	6.70E–01	2.75E–05*	1.79E–02*
Q13103	**Secreted phosphoprotein 2**	**SPP2**	1.10	0.000	1.40	–0.07	2.13E–04*	9.94E–01	2.07E–02*	1.86E–02*
P00746	**Complement factor D**	**CFD**	0.94	0.004	–0.60	0.03	1.32E–11*	4.93E–01	2.48E–02*	3.94E–02*
P35858	Insulin like growth factor binding protein acid labile subunit	IGFALS	1.30	–0.007	–0.31	0.02	4.10E–19*	1.38E–01	2.98E–01	4.14E–02*
P19320	Vascular cell adhesion molecule 1	VCAM1	1.04	0.000	1.17	–0.05	1.01E–04*	9.94E–01	3.84E–02*	5.21E–02
P00739	Haptoglobin-related protein	HPR	1.18	–0.002	–0.96	0.04	8.04E–08*	8.95E–01	2.04E–02*	6.42E–02
P05543	Serpin family A member 7	SERPINA7	0.86	0.006	0.72	–0.03	5.11E–07*	4.43E–01	4.40E–02*	1.44E–01
P01876	Immunoglobulin heavy constant alpha 1	IGHA1	0.95	0.004	–0.89	0.02	2.76E–06*	7.50E–01	2.04E–02*	2.52E–01

### Proteins related to functional outcome were identified in BMD patients

We then sought to determine whether any relationship exists between motor function and longitudinal protein signatures in patients with BMD. Five motor function performance metrics were investigated, 10-meter walk/run test velocity (10MWRTv), rise from floor (RFFv) velocity, climb/descend four stairs (4-SCv/4-SDv) velocity, and North Star Ambulatory Assessment (NSAA) score. A linear mixed effects model, with patient as random effect and one of each motor function performance metric as a fixed effect, revealed ten proteins, N-acetylmuramoyl-L-alanine amidase (PGLYRP2), gelsolin (GSN), tenascin-X (TNXB), serum amyloid P-component (APCS), complement C3 (C3), complement factor I (CFI), hemopexin (HPX), C4b-binding protein beta chain (C4BPB), plasminogen-like protein B (PLGLB1/ PLGLB2), and tetranectin (CLEC3B) which correlate with NSAA score in BMD. C4BPB and PGLYRP2 were also correlated with RFF (Table 2). Seven out of these ten proteins (C4BPB, HPX, CFI, C3, APCS, GSN, and PGLYRP2) are involved in the immune response.

### A2M trajectory changes with level of dystrophin expression in the TA of BMD patients

As several dystrophin restoring therapies are currently in clinical trials today, with both microdystrophin therapies and exon skipping therapies having gained accelerated FDA approval in recent years, we sought to investigate whether any of the observed protein trajectories were affected by dystrophin expression in BMD patients. Among the 34 BMD patients included in the present study, dystrophin expression in the TA was measured in12 patients, with expression ranging between 18% and 86% of that in healthy control skeletal muscle [14]. We identified, only alpha-2-macroglobulin (A2M) as being associated with dystrophin expression with significant FDR-values. A2M decreased faster over both time and age in patients with low dystrophin expression compared to patients with high expression of dystrophin (Table 3 and Fig. S5). This observation was in agreement with the age-related decline of A2M in DMD patients (Table 4), who either lack dystrophin expression in their muscles or display expression of only a few percent compared to healthy tissue. Interestingly, A2M did not decrease with age or time alone in the BMD patients (Table S3-S6).

**Table 3:** Proteins correlated with functional tests (ft) in BMD patients. Analysis was performed using a linear mixed effects model with the functional test (ft) as fixed effect and patient ID as random effect. Significant FDR-values are marked with an * and in grey

Uniprot ID	Protein description	Gene name	Functional test (ft)	β intercept	β ft	FDR intercept	FDR ft
P20851	C4b-binding protein beta chain	C4BPB	RFF	0,73	0,71	3,53E-13*	2,65E-02*
Q96PD5	N-acetylmuramoyl-L-alanine amidase	PGLYRP2	RFF	0,86	0,62	3,13E-14*	2,65E-02*
Q96PD5	N-acetylmuramoyl-L-alanine amidase	PGLYRP2	NSAA	0,88	0,22	5,07E-29*	7,73E-04*
P06396	Gelsolin	GSN	NSAA	0,95	0,25	1,19E-24*	1,16E-02*
P22105	Tenascin XB	TNXB	NSAA	0,86	0,46	5,51E-15*	1,36E-02*
P02743	Amyloid P component, serum	APCS	NSAA	1,10	-0,25	4,78E-25*	1,36E-02*
P01024	Complement C3	C3	NSAA	1,08	-0,21	7,30E-28*	2,03E-02*
P05156	Complement factor I	CFI	NSAA	1,08	-0,19	1,15E-27*	2,47E-02*
P02790	Hemopexin	HPX	NSAA	1,16	-0,21	7,01E-28*	2,80E-02*
P20851	C4b-binding protein beta chain	C4BPB	NSAA	1,15	-0,23	5,08E-25*	4,16E-02*
Q02325	Plasminogen-like protein B	PLGLB1/ PLGLB2	NSAA	0,96	0,19	1,09E-25*	4,52E-02*
P05452	Tetranectin	CLEC3B	NSAA	0,92	0,24	2,97E-21*	4,71E-02*

**Table 4:** Proteins correlated with dystrophin expression in BMD patients when accounting for either time or age. Analysis was performed using a linear mixed effects model with either time from first visit in years (time) or patient age (age), dystrophin expression in TA (dys%) as a fraction of expression in healthy tissue and their combination (time: dys % or age: dys %) as fixed effects and patient ID as random effect. Significant FDR-values are marked with an * and in grey

**Uniprot ID**	**Protein name**	**Gene name**	**β** **intercept**	**β** **time**	**β** **dys %**	**β** **time: dys %**	**FDR intercept**	**FDR** **time**	**FDR** **dys %**	**FDR** **time: dys %**
P01023	Alpha-2-macroglobulin	A2M	2.90	–0.041	–3.76	0.081	2.41E–05*	1.58E–02*	5.99E–02	2.08E–02*
**Uniprot ID**	**Protein name**	**Gene name**	**β** **intercept**	**β** **time**	**β** **dys %**	**β** **time: dys %**	**FDR intercept**	**FDR** **time**	**FDR** **dys %**	**FDR** **time: dys %**
P01023	Alpha-2-macroglobulin	A2M	1.20	–0.052	–0.40	0.10	4.38E–05*	2.54E–03*	7.11E–01	2.01E–03*

Abbreviations: DMD = Duchenne Muscular Dystrophy; BMD = Becker Muscular Dystrophy; NSAA = North Star Ambulatory Assessment; 10MWRTv = 10 m walk/run velocity; 4-SCv = 4-Stair Climb velocity; 4-SDv = 4-Stair Descend velocity; RFFv = Rise From Floor velocity. ^†^In-frame DMD variants were deletion of exons 3 to 18 and of exons 5 to 44. ^††^Both out-of-frame BMD variants were deletions of exons 3 to 7

## Discussion

Several studies [1, 33, 34] have assumed the translatability of DMD biomarker candidates to BMD because of the disease similarities and shared genetic backgrounds. However, the variations in disease onset, age range and disease severity argument for a thorough analysis of the blood proteome and a non-hypothesis-driven approach for the identification of biomarker candidates within the context of the dystrophinopathy spectrum. Importantly, the low BMD disease prevalence and the large phenotypic variation complicate biomarker studies. A unique set of 107 BMD samples, collected within two prospective natural history studies 48 DMD samples, have been analysed by mass spectrometry to explore the serum proteome of the dystrophinopathy disease spectrum.

Our study identified one protein, PKM, which changes with age in BMD patients with both altered slope and trajectory compared with DMD patients. Among the proteins with an altered association with age between DMD and BMD were several muscle-related proteins (CKM, PKM, MYOM3, PI16, LDHB, and GSN). CKM, PKM, and LDHB are enzymes involved in energy production and transduction in tissues with high energy demands like the muscle. MYOM3, GSN and PI16 are involved in muscle function, cytoskeletal organization and muscle regeneration. CKM has previously been proposed as a monitoring and surrogate endpoint biomarker in DMD [35]. However, previous studies have failed to identify a relationship between CKM and motor function performance tests in BMD [19]. This was confirmed in our study. Other disease progression biomarker studies in DMD have identified LDHB [8, 32] and FGG [8, 32] to be related to functional outcomes or clinical milestones, such as loss of ambulation. In contrast, our study revealed proteins that are part of the complement cascade, to change with NSAA score, suggesting an increase in inflammation in BMD patients as the disease progresses. Two secreted proteins expressed in muscle, GSN and CLEC3B, were also found to be correlated with motor function. However, since muscle-derived leakage proteins are commonly found at low concentrations in serum, and the rate of muscle cell damage in BMD is assumed to be much lower than that in the DMD, this study potentially missed leakage proteins due to insufficient penetration into serum proteome. Of the proteins correlated with function in BMD, four proteins were also found to change with age in DMD patients: GSN, TNXB, C4BPB and APCS. It is interesting to note that only two of the inflammatory markers– GSN and C4BPB– are found to change with age in DMD. This may be because nearly all DMD participants were taking corticosteroids, while all BMD participants were steroid-naïve. Ideally, a comparison between steroid naïve and treated DMD patients should be made. However, in view of the highly recommended initiation of treatment around the age of 4–5, this will likely not be feasible in the current population.

A previous proteomic study revealed that serum levels of transport proteins, such as hemopexin (HPX), are elevated in muscle biopsies from DMD patients [36]. Interestingly, in our study, HPX was found to be elevated in serum of BMD patients with reduced performance in NSAA score. The same study [36] reported that extracellular matrix proteins, such as A2M and FGG, were elevated in DMD muscle compared to BMD muscle. It is therefore noteworthy that, our study identified serum A2M levels to be elevated in DMD patients compared to BMD, patients. In addition, serum A2M levels decreased faster over time in relation to the amount of dystrophin expressed in BMD muscle. A2M is a versatile protease inhibitor which provides protection against systemic inflammation and aids clearance of damaged extracellular proteins [37]. The protein is produced and secreted by the liver and macrophages [38], and has been reported to be negatively associated with age in healthy children [38, 39]. It can therefore be debated whether the age-association of A2M in our study was due to disease progression or patient aging. Future validation of A2M in both dystrophinopathy patients and age-matched healthy individuals is therefore needed.

The samples from DMD patients are collected between the ages of 7 and 24 years, whereas the samples from BMD patients are collected between the ages of 20 and 68 years due to the scarcity of patients. Although samples are collected within a wide age range, the value of identifying biomarker candidates across the dystrophinopathy spectrum will provide knowledge about mild disease phenotypes that will benefit the BMD population and most likely aid future characterisation of DMD patients treated with gene therapies, designed to convert the DMD phenotype into a milder phenotype. A limitation of this study is the relatively short follow-up of three years in slowly progressive disease like BMD. However, in a subgroup of the current cohort, we previously demonstrated a significant decline in NSAA scores and 10MWRv and an increase in muscle fat-fraction measured with magnetic resonance imaging (MRI) within 24 months [40]. These alterations are most likely associated with the replacement of muscle cells with fat and molecular changes in tissue composition that are likely to be reflected in the blood. Increasing the duration of follow-up in natural history study protocols coincides with patients being less likely to participate because of the burden of hospital visits and functional tests. Furthermore, the medical ethical committee is less likely to approve a protocol with a long follow-up given the absence of personal benefit of partaking in a natural history study. When a long follow-up is desired, for instance, to enable linking biomarkers to the occurrence of clinical milestones, integration of biobanking and standardized functional tests in routine clinical care is required. Despite the increasing number of ongoing DMD and BMD clinical studies, collecting age-matched control samples still remains a challenge. The reluctance of parents to allow collection of samples from otherwise healthy children and the limited volume of blood that can be collected without a negative impact on the individual limits the possibility to collect healthy control samples. In the case of BMD the patient rarity and the wide age variation at sampling makes it challenging to collect age-matched samples from healthy individuals. However, to evaluate the effect of aging and validate the biomarkers presented in this paper, future studies have to include age-matched controls e.g. the involvement of healthy family members that are more likely to participate and commit to such clinical studies. In spite of these limitations we believe that our study fills in a knowledge gap regarding the differences in disease phenotypes defined as in DMD and BMD, but in essence part of the dystrophinopathy spectrum. The value of comparing serum composition in the context of DMD and BMD will benefit not only the clinical management of BMD but also future DMD patients treated with gene therapies. DMD children currently treated with gene therapies will most likely present new phenotypes more similar to the ones encountered in the existing BMD population due to the expression of an altered but functional dystrophin protein.

## Conclusions

These results revealed 10 potential biomarkers reflecting disease progression in BMD and suggest that BMD progression monitoring biomarkers are more likely found to be proteins involved in the innate immune response (C4BPB, PGLYRP2, GSN, CFI, C3, HPX and APCS), extracellular matrix organization (PLGLB1/ PLGLB2 and TNXB) and hemostasis (CLEC3B), than among muscle leakage proteins, which have been associated with disease progression in DMD patients. Our study highlights similarities and differences in disease progression related proteomic changes between DMD and BMD patients, indicating that the translation of disease progression biomarkers from DMD to BMD may not be as straightforward as previously assumed. In addition, A2M was associated with dystrophin expression in the tibialis anterior muscle making it a potential pharmacodynamic biomarker if further validated.

## Electronic supplementary material

Below is the link to the electronic supplementary material.

Supplementary Material 1

Supplementary Material 2

## Data Availability

All data can be accessed through https://panoramaweb.org/r5jRCR.url.
