# Supplementary material for: Contrasting Becker and Duchenne muscular dystrophy serum biomarker candidates by using data independent acquisition LC-MS/MS
Source: Skelet Muscle. 2025 Jun 7;15:15. doi: 10.1186/s13395-025-00385-3 (PMC12144774; doi:10.1186/s13395-025-00385-3)
Supplement: Supplementary file 1 — Supplementary Material 1 [file 13395_2025_385_MOESM1_ESM.pdf]

Supporting information for:

Contrasting Becker and Duchenne Muscular Dystrophy serum biomarker candidates by using Data Independent Acquisition LC-MS/MS

Camilla Johansson[1], Esther Schrama[3], David Kotol[2], Andreas Hober[3], Zaïda Koeks[3], Nienke van de Velde[3], Jan J G M Verschuuren[3], Erik H. Niks[3], Fredrik Edfors[2], Pietro Spitali[4], Cristina Al-Khalili Szigyarto[1,2]*

1. Department of Systems biology, School of Chemistry, Biology and Health, KTH Royal Institute of Technology, Stockholm, Sweden.
2. Science for Life Laboratory, School of Chemistry, Biology and Health, KTH Royal Institute of Technology, Stockholm, Sweden.
3. Department of Neurology, Leiden University Medical Center, Leiden, The Netherlands.
4. Department of Human Genetics, Leiden University Medical Center, Leiden, The Netherlands.

*Corresponding author

Table S1. Protein standards used for the MS analysis

Stable isotope labeled standards	Protein description	Protein	Amount/sample [pmol]
HPRR2370122	carbonic anhydrase 3	CA3	0.049
HPRR1770039	collagen type I alpha 1 chain	COL1A1	0.131
HPRR670085	afamin	AFM	0.108
HPRR140864	serpin family A member 10	SERPINA10	0.008
HPRR4220446	fibrinogen gamma chain	FGG	0.290
HPRR320021	serpin family A member 1	SERPINA1	3.679
HPRR1420044	nestin	NES	0.172
HPRR2190016	myosin light chain 3	MYL3	0.048
HPRR2380028	electron transfer flavoprotein subunit alpha	ETFA	0.041
HPRR2380027	electron transfer flavoprotein subunit alpha	ETFA	0.033
HPRR2440507	malate dehydrogenase 2	MDH2	0.128
HPRR2440504	malate dehydrogenase 2	MDH2	0.028
HPRR2440506	malate dehydrogenase 2	MDH2	0.044
HPRR2960051	microtubule associated protein 4	MAP4	0.046
HPRR3050240	troponin T3, fast skeletal type	TNNT3	0.142
HPRR4290158	lactate dehydrogenase B	LDHB	0.055
HPRR2380029	electron transfer flavoprotein subunit alpha	ETFA	0.106
HPRR2380026	electron transfer flavoprotein subunit alpha	ETFA	0.163
HPRR350060	joining chain of multimeric IgA and IgM	JCHAIN	1.931
HPRR4030673	vitronectin	VTN	0.806

Table S2. LC-gradient for SRM-MS

Time	Flow [µl/min]	%B
0	3	1
0.75	3	1
30	3	30
30.1	3	95
30.5	3	95
31	3	1
31.5	3	95
32	3	95
32.5	3	1
33	3	95
33.5	3	95
33.6	3	1
35	Stop Run	

Table S3. Proteins correlated with age in DMD patients using a linear mixed effects model. Patient age at visit was kept as fixed effect and patient ID as random effect.

Uniprot ID	Protein description	Gene name	*β* Intercept	*β* Age	*P*-value Intercept	*P*-value Age	FDR Intercept	FDR Age
P00746	Complement factor D	CFD	0,35	0,033	1,03E-03	7,08E-06	1,49E-03	5,90E-04
P80108	Phosphatidylinositol-glycan-specific phospholipase D	GPLD1	0,13	0,038	3,25E-01	4,74E-06	3,50E-01	5,90E-04
Q5VTT5	Myomesin 3	MYOM3	5,63	-0,252	3,97E-08	6,24E-06	1,18E-07	5,90E-04
P06732	Creatine kinase, M-type	CKM	5,80	-0,264	2,66E-07	2,27E-05	6,45E-07	1,04E-03
P07195	Lactate dehydrogenase B	LDHB	6,15	-0,271	6,71E-08	1,68E-05	1,91E-07	1,04E-03
Q9Y6R7	IgGFc-binding protein	FCGBP	0,09	0,050	6,39E-01	2,50E-05	6,63E-01	1,04E-03
P02679	Fibrinogen gamma chain	FGG	9,52	-0,423	3,03E-07	3,89E-05	7,21E-07	1,39E-03
P51884	Lumican	LUM	1,76	-0,044	1,44E-12	6,48E-05	1,33E-11	1,80E-03
Q9UGM5	Fetuin-B	FETUB	2,49	-0,069	7,92E-12	6,47E-05	6,18E-11	1,80E-03
P01023	Alpha-2-macroglobulin	A2M	2,34	-0,046	2,99E-16	7,21E-05	1,49E-14	1,80E-03
P00739	Haptoglobin-related protein	HPR	0,21	0,034	1,62E-01	1,01E-04	1,82E-01	2,29E-03
P14618	Pyruvate kinase PKM	PKM	2,93	-0,111	1,11E-07	1,15E-04	2,87E-07	2,40E-03
P02743	Amyloid P component, serum	APCS	0,33	0,033	1,62E-02	1,61E-04	2,07E-02	2,90E-03
P11021	Heat shock protein family A member 5	HSPA5	2,20	-0,063	3,06E-10	1,62E-04	1,39E-09	2,90E-03
P22105	Tenascin XB	TNXB	2,25	-0,080	2,63E-08	1,82E-04	8,54E-08	3,04E-03
P01031	Complement C5	C5	0,73	0,015	1,24E-14	2,17E-04	3,10E-13	3,39E-03
P01876	Immunoglobulin heavy constant alpha 1	IGHA1	0,06	0,027	6,19E-01	2,94E-04	6,44E-01	4,33E-03
P06396	Gelsolin	GSN	0,45	0,018	2,39E-06	4,63E-04	4,99E-06	6,43E-03
P02675	Fibrinogen beta chain	FGB	5,21	-0,201	1,98E-06	6,36E-04	4,23E-06	8,37E-03
Q04756	Hepatocyte growth factor activator	HGFAC	1,42	-0,026	6,05E-14	1,22E-03	9,45E-13	1,53E-02
P17066	Heat shock protein family A member 6	HSPA6	2,29	-0,066	2,74E-08	1,31E-03	8,70E-08	1,56E-02
Q96IY4	Carboxypeptidase B2	CPB2	0,65	0,024	1,77E-06	1,56E-03	3,81E-06	1,77E-02
P20851	C4b-binding protein beta chain	C4BPB	0,74	0,015	4,91E-11	1,68E-03	2,79E-10	1,83E-02
P01011	Alpha-1-antichymotrypsin	SERPINA3	0,62	0,024	2,13E-05	2,95E-03	3,95E-05	2,95E-02
P0DOX2	Immunoglobulin alpha-2 heavy chain	IGA2	-0,09	0,030	5,68E-01	2,83E-03	5,95E-01	2,95E-02
Q9UHG3	Prenylcysteine oxidase 1	PCYOX1	0,61	0,038	3,44E-03	3,68E-03	4,81E-03	3,54E-02
P05543	Serpin family A member 7	SERPINA7	1,65	-0,027	5,52E-13	4,28E-03	6,00E-12	3,96E-02
Q6UXB8	Peptidase inhibitor 16	PI16	4,14	-0,126	9,45E-07	4,98E-03	2,07E-06	4,45E-02
P35527	Keratin, type I cytoskeletal 9	KRT9	1,38	-0,034	1,47E-09	5,18E-03	5,67E-09	4,46E-02

Table S4. Proteins correlated with age in BMD patients using a linear mixed effects model. Patient age at visit was kept as fixed effect and patient ID as random effect.

Uniprot ID	Protein description	Gene name	*β* Intercept	*β* Age	*P*-value Intercept	*P*-value Age	FDR Intercept	FDR Age
P14618	Pyruvate kinase PKM	PKM	1,59	-0,013	2,66E-14	1,23E-04	1,39E-13	1,76E-02
P17936	Insulin-like growth factor-binding protein 3	IGFBP3	1,45	-0,011	5,75E-17	1,41E-04	4,49E-16	1,76E-02

Table S5. Proteins correlated with time from first enrollment in study in DMD patients using a linear mixed effects model. Time from enrollment, in years, was kept as fixed effect and patient ID as random effect.

**Uniprot ID**	**Protein description**	**Gene name**	***β* Intercept**	***β* Age**	***P*-value Intercept**	***P*-value Age**	**FDR Intercept**	**FDR Age**
Q5VTT5	Myomesin 3	MYOM3	2,21	-0,383	2,45E-06	3,43E-05	2,89E-06	8,58E-03
P06732	Creatine kinase, M-type	CKM	2,18	-0,358	7,68E-06	2,52E-04	8,84E-06	1,28E-02
P07195	Lactate dehydrogenase B	LDHB	2,48	-0,392	7,87E-07	2,56E-04	9,41E-07	1,28E-02
P80108	Phosphatidylinositol-glycan-specific phospholipase D	GPLD1	0,68	0,038	3,00E-09	1,87E-04	4,21E-09	1,28E-02
Q9Y6R7	IgGFc-binding protein	FCGBP	0,84	0,048	3,32E-06	1,78E-04	3,90E-06	1,28E-02
P00739	Haptoglobin-related protein	HPR	0,72	0,034	2,11E-07	4,93E-04	2,57E-07	2,05E-02

Table S6. Proteins correlated with time from first enrollment in study in BMD patients using a linear mixed effects model. Time from enrollment, in years, was kept as fixed effect and patient ID as random effect.

**Uniprot ID**	**Protein description**	**Gene name**	***β* Intercept**	***β* Age**	***P*-value Intercept**	***P*-value Age**	**FDR Intercept**	**FDR Age**
P03951	Coagulation factor XI	F11	1,24	-0,11	1,22E-18	2,75E-13	1,85E-18	6,88E-11
P68133	Actin, alpha skeletal muscle	ACTA1	0,89	0,052	3,71E-23	5,12E-05	6,71E-23	6,40E-03
P27918	Properdin	CFP	1,18	-0,021	1,01E-29	4,17E-04	3,11E-29	3,47E-02
P35858	Insulin-like growth factor-binding protein complex acid labile subunit	IGFALS	1,02	-0,016	3,87E-29	8,00E-04	1,10E-28	5,00E-02

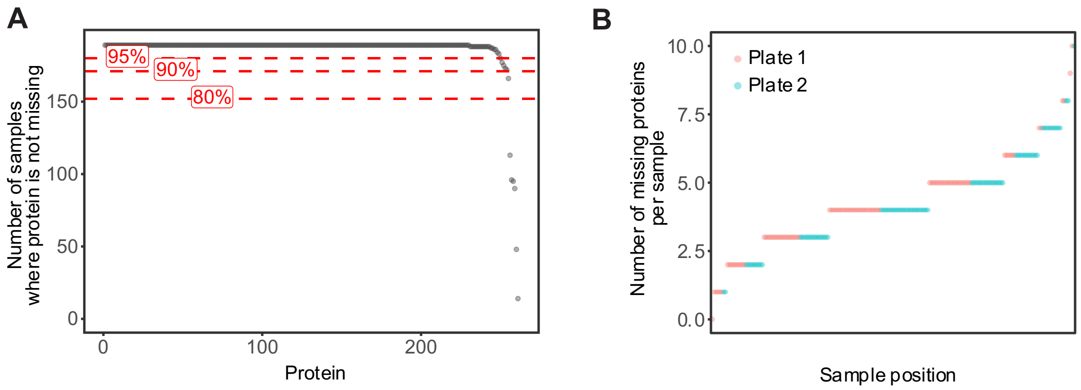

Figure S1. Filtering of proteins based on the number of missing values (missing proteins). A. Filter cut-off for missing values per proteins. Data was filtered based on 95% cut-off. **B.** Number of missing proteins per sample, coloured by plate to discover batch effects.

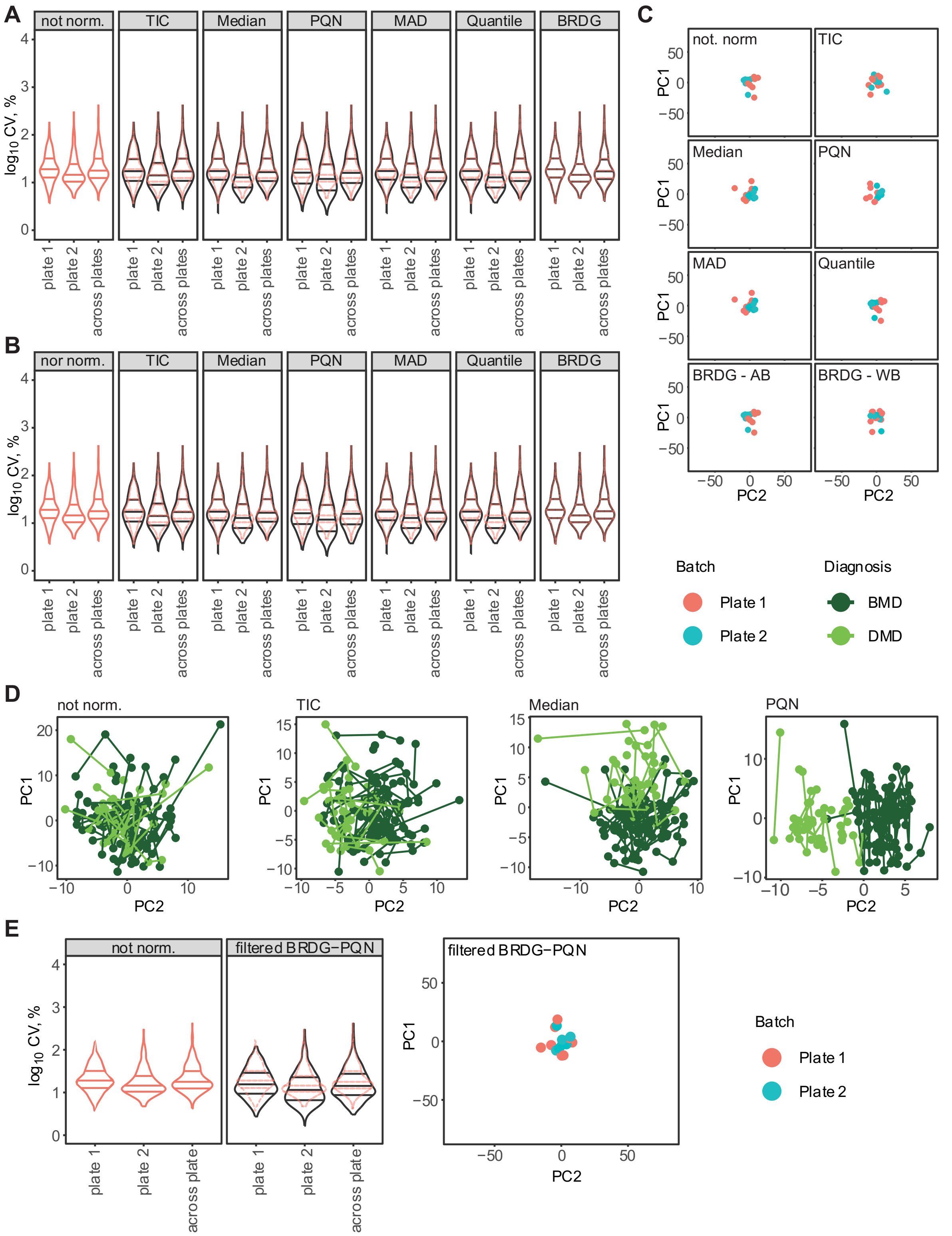

Figure S2. Investigating different data normalisation methods for reducing batch and plate effects. A-B. CV% for all proteins in QC samples before (not norm.) and after applying different data normalisation methods, **A.** normalized for each plate separately or. **B.** across both plates. Red dotted lines show an overlay of the violin plots for data before normalization. **C.** Principal Component Analysis of QC samples before and after data normalisation, coloured by plate. **D.** Principal component analysis on whole cohort (excluding QC samples) before (not norm.) and after applying different normalization methods. **E.** CV before and after final normalisation method, where data was first bridge normalised followed by PQN.
TIC: Total Ion Current. Median: Median normalisation. PQN: Probabilistic Quotient Normalisation. MAD: Median Absolute Deviation. Quantile: Quantille normalisation. BRDG: Bridge normalisation [1].

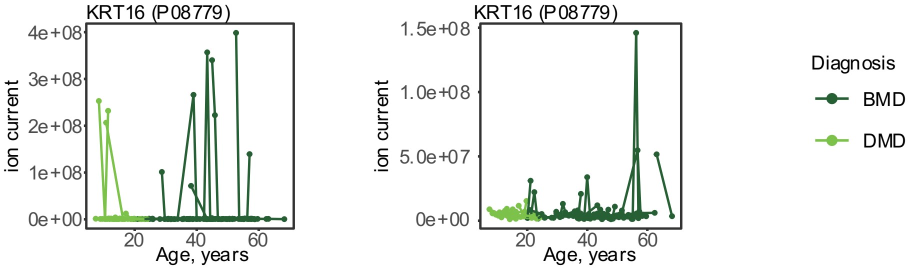

Figure S3. Examples of proteins displaying extreme outlier behaviors.

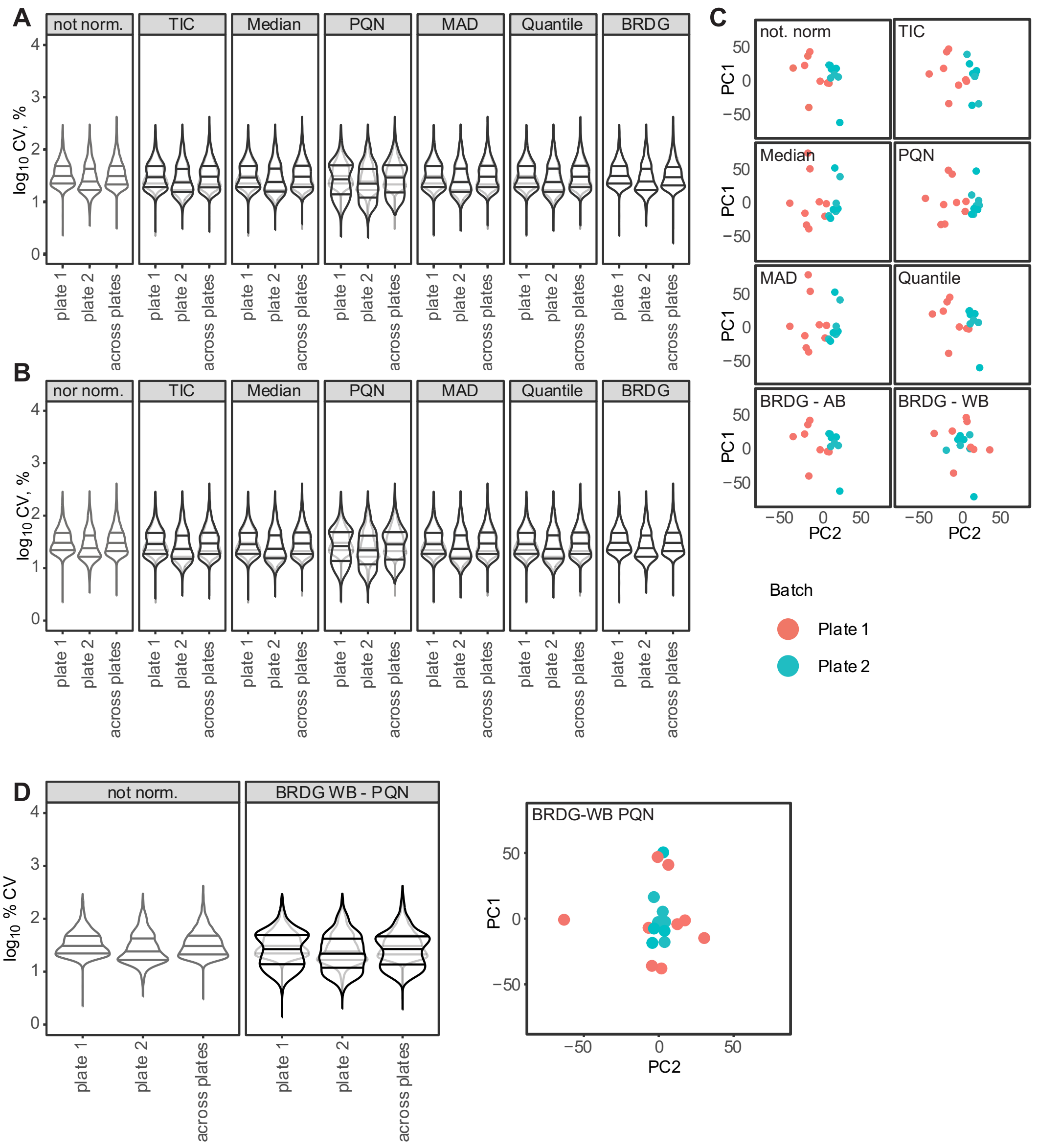

Figure S4. Investigating different data normalisation methods for reducing batch and plate effects on peptide level. A-B. CV% for all proteins in QC samples before (not norm.) and after applying different data normalisation methods, **A.** normalized for each plate separately or. **B.** across both plates. Red dotted lines show an overlay of the violin plots for data before normalization. **C.** Principal Component Analysis of QC samples before and after data normalisation, coloured by plate. **D.** CV before and after final normalisation method, where data was first bridge normalised followed by PQN.

TIC: Total Ion Current. Median: Median normalisation. PQN: Probabilistic Quotient Normalisation. MAD: Median Absolute Deviation. Quantile: Quantille normalisation. BRDG: Bridge normalisation [1].

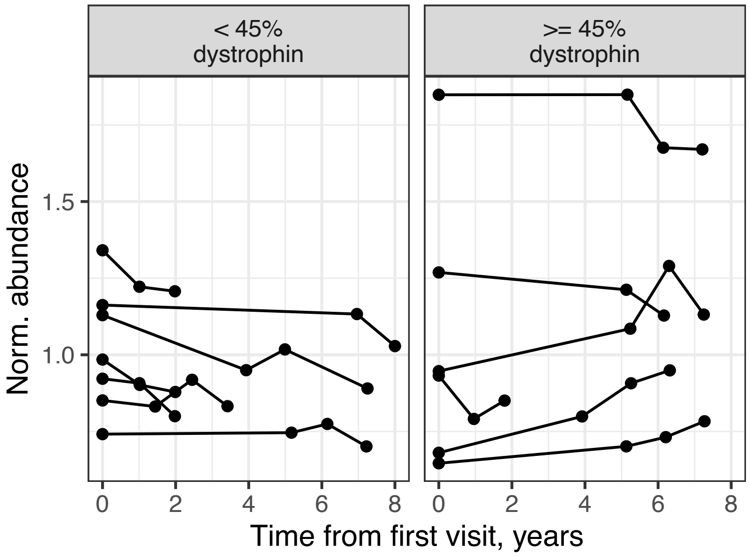

Figure S5. Time-related trajectories for protein A2M in BMD patients with < 45% dystrophin expression or ≥ 45% dystrophin expression in TA.

References:

1. Wulff, J.E. and M.W. Mitchell, *A comparison of various normalization methods for LC/MS metabolomics data.* Advances in Bioscience and Biotechnology, 2018. **9**(08): p. 339-351.
